# Graphene-assisted ultra-compact polarization splitter and rotator with an extended bandwidth

**DOI:** 10.1038/s41598-017-12536-8

**Published:** 2017-09-22

**Authors:** Tian Zhang, Xianmin Ke, Xiang Yin, Lin Chen, Xun Li

**Affiliations:** 1 Wuhan National Laboratory for Optoelectronics, Huazhong University of Science and Technology, Wuhan, 430074 China; 2 State Key Laboratory of Information Photonics and Optical Communications, Beijing University of Posts and Telecommunications, Beijing, 100876 China

## Abstract

The high refraction-index contrast between silicon and the surrounding cladding makes silicon-on-insulator devices highly polarization-dependent. However, it is greatly desirable for many applications to address the issue of polarization dependence in silicon photonics. Here, a novel ultra-compact polarization splitter and rotator (PSR), constructed with an asymmetrical directional coupler consisting of a rib silicon waveguide and a graphene-embedded rib silicon waveguide (GERSW), on a silicon-on-insulator platform is proposed and investigated. By taking advantage of the large modulation of the effective refractive index of the TE mode for the GERSW by tuning the chemical potential of graphene, the phase matching condition can be well satisfied over a wide spectral band. The presented result demonstrates that for a 7-layer-graphene-embedded PSR with a coupling length of 11.1 μm, a high TM-to-TE conversion efficiency (>−0.5 dB) can be achieved over a broad bandwidth from 1516 to 1602 nm.

## Introduction

Integrated photonic devices built on silicon-on-insulator (SOI) have been attractive for their compatibility with mature, complementary metal-oxide-semiconductor-compatible technologies^1^. While the high-refractive index contrast between the silicon and surrounding cladding has the advantage of constructing photonic devices with a compact footprint, it inevitably poses a significant challenge to handling the polarization dependence. To address this issue, a polarization diversity circuit that consists of polarization beam splitters and rotators is highly desirable^2–6^. A polarization beam splitter can efficiently split one input beam with two orthogonal polarization states into two output beams with different polarization states^3,5^, while a polarization beam rotator can rotate the polarization of input beam by 90°^4^. For many applications, in the rest of an optical circuit only a certain polarization state can be guided. Therefore, it is highly desirable to develop polarization management devices that are capable of rotating one of the two polarization states to the orthogonal state so that merely one polarization state must be processed in the rest of the optical circuit. Recently, a type of polarization splitter and rotator (PSR) technology has been intensively proposed to achieve this purpose. With this technology, one polarization state of input beam is converted to the orthogonal polarization state using a directional coupler. Meanwhile, the orthogonal polarization state of input beam is much unaffected and output through the input waveguide because no coupling occurs in the region of the directional coupler^7–12^. Therein, a PSR using an asymmetrical directional coupler (ADC) composed of two waveguides with broken symmetry for the waveguide cross section has attracted much research attention^8,12^. However, the most apparent drawback of the scheme is that it can merely operate within a limited bandwidth, as the phase-matching condition should be precisely satisfied for an ADC. While promising steps have been taken to broaden the operation bandwidth by utilization of tapered^9,10^ and taper-etched^7,11^ ADCs, it is generally accompanied with a relatively large footprint, which is against the high-density integration on the SOI platform.

Graphene, a two-dimensional single layer of carbon atoms arranged in a hexagonal lattice, has attracted tremendous attention in recent years due to its unique optical and electrical properties. Owing to the large and flexible tunability of graphene by bias voltage and chemical doping, graphene is emerging as an attractive material for the development of highly efficient optoelectronics devices including electro-absorption modulators^13–16^ and electro-refractive modulators^17^. A recent study has demonstrated that graphene will have different influences on the modal characteristics of silicon waveguides for different polarization states when graphene is involved in a dielectric waveguide, thus offering huge capability to address the issue of strong polarization dependence^5,18–20^.

In this article, we propose and numerically demonstrate a novel PSR using a graphene-embedded silicon rib ADC. When graphene is horizontally embedded into a rib silicon waveguide (RSW), the effective refractive index (ERI) of the TE mode undergoes a significant variation by tuning the chemical potential of graphene via bias voltage. Consequently, the phase-matching condition for the ADC can be satisfied over a wide spectral band by properly choosing the variation range of the chemical potential of graphene, and hence the operation bandwidth of the PSR can be significantly enlarged.

## Results and Discussion

**Figure 1:**
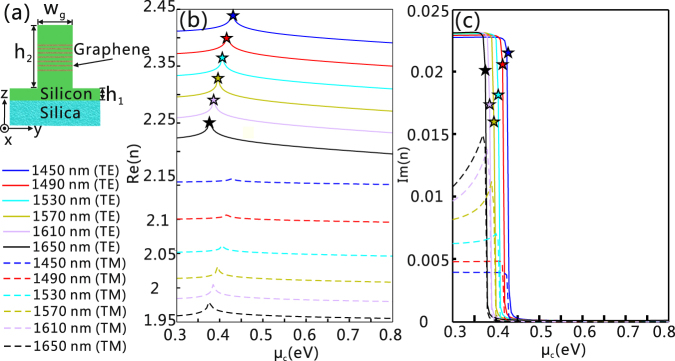
The modal characteristics of the GERSW. (**a**) Schematic cross section of the GERSW. (**b**,**c**) The modal characteristics for both the TE and TM modes in the GERSW at different wavelengths as a function of chemical potential of graphene. The simulation work is conducted by a finite difference eigenmode solver with a commercial software Lumerical MODE Solutions: (**b**) The real parts of the effective refractive index [Re(n)] and (**c**) imaginary parts of the effective refractive index [Im(n)]. The solid and dashed lines represent the modal characteristics for the TE and TM modes, respectively. The peak positions for the Re(n) of the TE mode are marked by stars in (**b**) and (**c**). Here, *w*_g_ = 300 nm, *h*_1_ = 90 nm, *h*_2_ = 212.38 nm, the layer of graphene is 7, and the gap distance between adjacent graphene layers is 20 nm. Undoped amorphous silicon is chosen to construct the GERSW. In the simulation, non-uniform mesh grids are used, and the maximum mesh grids for both the silicon and graphene layers are set at 4 nm along the y-direction, and 5, and 0.085 nm along the z-direction, respectively.

A graphene-embedded rib silicon waveguide (GERSW) is shown in Fig. 1(a). The relative permittivities of silicon and silica layers are 12.04 and 2.09, respectively^21^, and the optical property of the graphene layer has been provided in the Methods section. The chemical potential of the graphene multilayer has a stronger effect on the modal characteristics of the TE mode than that of the TM mode [shown in Fig. 1(b) and (c)]. For example, the difference between the maximum and the minimum values for the Re(*n*) of the TE mode at 1450 nm is 0.057, while that of the TM mode is only 0.024. In the wavelength range of interest, the modal characteristics for both the TE and TM modes presents a sharp peak due to the dramatic change of the graphene’s conductivity around *μ*_c_ = 0.4 eV^22^. It should be noted that the propagation losses of the TE modes at the peaks in Fig. 1(b), corresponding to the values of Im(*n*) denoted by the star marks in Fig. 1(c), are much larger than that of the TE mode as a slightly larger *μ*_c_ is chosen.

**Figure 2:**
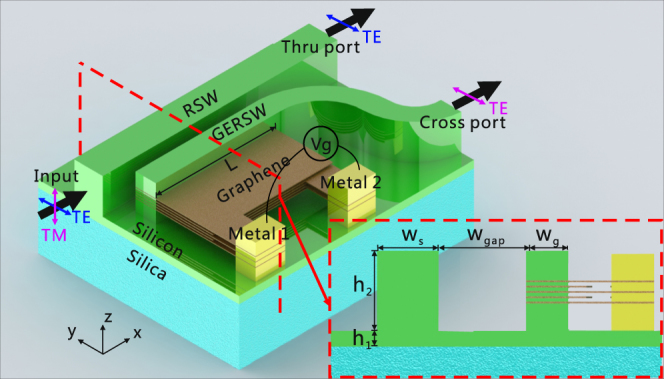
Schematic configuration of the proposed PSR. The cross section for the coupling region is shown in the lower-right corner. In this work, *w*_s_ and *h*_1_ are fixed at 500 nm and 90 nm, respectively. The RSW is as high as the GERSW.

Bearing these points in mind, we propose to construct a PSR consisting of a GERSW and a RSW, as shown in Fig. 2. The gap separation (*w*_gap_) between the two waveguides is unchanged in the ADC region, and an S-bend waveguide is connected to the GERSW. Metal 1 and Metal 2 act as two electrodes. The modal field of the TE mode is strongest in the centre of the RSW. Thus, in order to increase the influence on the modal characteristic of the TE mode, the graphene layers must be inserted into the centre of the RSW to maximize the graphene-light interaction. Further, the modal characteristic of the TE mode objects to a larger influence if more graphene layers are used^18,22^. In our work, we have chosen an odd number of graphene layers to illustrate the performance of the PSR. We emphasize that an even number of graphene layers applies to the design of the PSR as well, and the resultant PSR is expected to present a similar performance. We have noted a series of studies on the utilization of an even number of graphene layers for the construction of various graphene-based optoelectronics devices on a silicon platform, including silicon waveguide modulator^14,23^ and polarizer^18^. The odd number of graphene layers are extended to contact the Metal 1, and the remaining layers contact the Metal 2. The utilization of undoped, amorphous silicon ensures the formation of capacitor effect between the graphene layers contacted to the metallic electrodes^23^. Thus, the chemical potential of the graphene layers could be controlled by the bias voltage applied on these two metal electrodes. The chemical potential of graphene, *μ*_c_, can be tuned by the gate voltage applied on graphene, ${V}_{g}$, through $|{u}_{c}|=\hslash {v}_{F}\sqrt{\pi {\varepsilon }_{d}{\varepsilon }_{0}|{V}_{g}-{V}_{Dirac}|/({h}_{d}e)}$ ^22^, where ${v}_{F}$≈1 × 10^6^ m/s is the Fermi velocity, and $|{V}_{g}-{V}_{Dirac}|$ would be the applied gate voltage because ${V}_{Dirac}$ is close to zero; ${\varepsilon }_{0}$ and ${\varepsilon }_{d}$ are the permittivity of air and the relative permittivity of silicon, respectively; and ${h}_{d}$ and $e$ are the thickness of silicon layer between the adjacent graphene layers and the electron charge, respectively. In the design of the two metallic electrodes, a certain distance between the silicon waveguides and electrodes should be kept to effectively eliminate the influence of the electrodes on the modal profile^13,18^. For the fabrication of the PSR, we can first prepare two shadow masks for the RSW and two metal electrodes. By properly arranging the order of the two masks, choosing the deposition methods, and transferring the graphene layers (etched in an “L” shape by a focused ion beam without the utilization of masks) from the copper foil, the presented PSR, at least in principle, might be fabricated^5^. In order to enable an efficient conversion between two orthogonal fundamental modes, the phase-matching condition is required. Considering that the modal characteristics for the TE mode in the GERSW can be changed with a broader range than that for the TM mode by tuning the chemical potential of graphene, the TE mode in the GERSW and the TM mode in the RSW are employed to satisfy the phase-matching condition. In this case, the largest operation bandwidth might be achieved because the phase-matching condition can be satisfied within a wide spectral band. In addition, we employed rib waveguides to increase the vertical asymmetry in the waveguide cross section, which is helpful for the mode conversion between the fundamental TE and TM modes. By optimizing the widths of two rib waveguides and the coupling length (*L*), the input TM mode from the RSW could be efficiently converted into the TE mode in the GERSW and output from the cross port, while the input TE mode is rather unaffected and directly output from the thru port.

**Figure 3:**
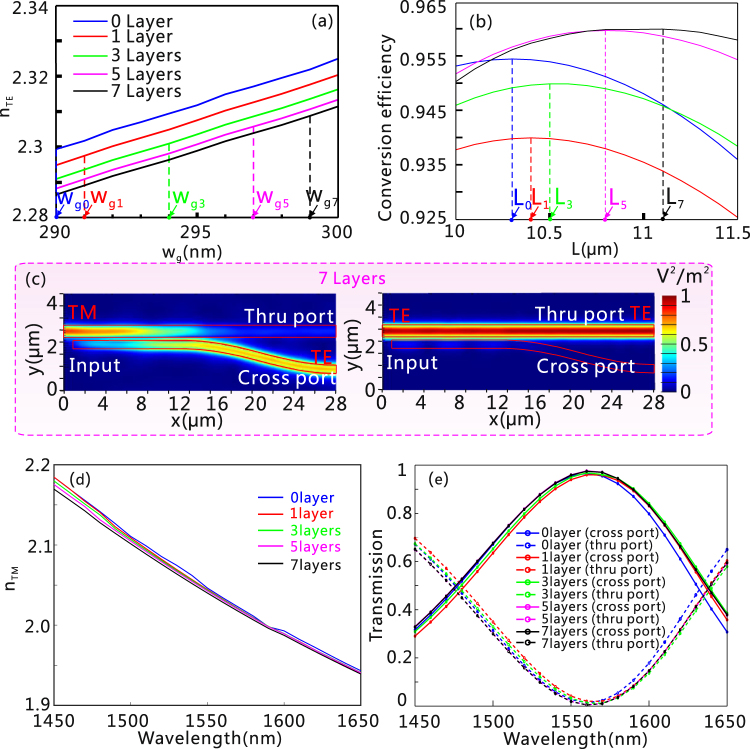
The conversion efficiency of the GERSW-based PSR with different number of graphene layer. (**a**) The ERI of the fundamental TE mode for the GERSW as a function of *w*_g_ with 0 (blue solid line), 1 (red solid line), 3 (green solid line), 5 (pink solid line), and 7 (black solid line) layers of graphene. In order to satisfy the phase-matching condition between the TM mode in the RSW and the TE mode in the GERSW, the widths of the GERSW for these five cases are set at *w*_g0_ = 290 nm, *w*_g1_ = 291 nm, *w*_g3_ = 294 nm, *w*_g5_ = 297 nm, *w*_g7_ = 299 nm, respectively. (**b**) Conversion efficiency as a function of the coupling length (*L*) with the optimum widths of the GERSW. The vertical dashed lines represent the optimum coupling length (*L*_0_ = 10.3 μm, *L*_1_ = 10.4 μm, *L*_3_ = 10.5 μm, *L*_5_ = 10.8 μm, and *L*_7_ = 11.1 μm, respectively), where the maximum conversion efficiency is achievable for the five cases. (**c**) Field distribution of light, |E|^2^, propagating along the 7-layer-graphene embedded PSR for the TE mode (left panel) and the TM mode (right panel) input. (**d**) Wavelength dependence on the ERI of the fundamental TM mode for the GERSW with 0 (blue solid line), 1 (red solid line), 3 (green solid line), 5 (pink solid line), and 7 (black solid line) layers of graphene. For the cases of 1, 3, 5, and 7 layers of graphene, the silicon layers above the top graphene layer are 105, 85, 65, and 45 nm, respectively. (**e**) Wavelength dependence of the PSR with the TM mode input. Each sheet of graphene used for the GERSW has the same length as that of the directional coupler, and the width four times as large as the width of the GERSW. Such a gap separation between the GERSW and the metallic electrodes is sufficiently large to avoid the influence of the metallic electrodes on the modal characteristics of the GERSW itself. Here, the wavelength is *λ* = 1550 nm, *w*_gap_ = 150 nm, and the height of the RSW and GERSW in these five cases are *h*_20_ = 210 nm, *h*_21_ = 210.3 nm, *h*_23_ = 211 nm, *h*_25_ = 211.7 nm, and *h*_27_ = 212.4 nm, respectively. In (**a**) and (**e**), the mesh grids used for the graphene and silicon layers are the same as those in Fig. 1. In (**b**–**d**), the simulation of the propagation characteristic is conducted by finite difference time domain (FDTD) simulation with non-uniform mesh grids. The maximum mesh grids for the silicon are set to be 15, 4, and 5 nm along the x, y, and z-directions, respectively, while those for graphene layers are 15, 4, and 0.085 nm along the x, y, and z-directions, respectively. All the mesh grids used in the following modeling work are the same as those in (**b**–**d**).

Next, we consider the performance of the designed PSR at *λ* = 1550 nm. In order to find the optimum width of the GERSW (*w*_g_) that satisfies the phase-matching condition, the ERI of the fundamental TE mode in the GERSW as a function of *w*_g_ has been numerically calculated [shown in the Fig. 3(a)]. The initial chemical potential of graphene is set at 0.5 eV considering that the value of Re(*n*) for the TE mode is roughly located in the middle of the tuning range [see Fig. 1], which assists in facilitating the tunability of the operation frequency of PSR. In our work, the gap separation, *w*_gap_, is chosen as 150 nm to build a compact directional coupler with acceptable fabrication difficulty. The coupling length, associated with the device size, can be further decreased by use of a smaller gap separation but at the cost of remarkably increased fabrication difficulty. In the design, the total thickness of the silicon layer in the GERSW is set at a constant value of 210 nm. The height of the GERSW, *h*_2_, is increased as the number of graphene increases. To make the two waveguides have the same height, the height of the RSW, *h*_2_, increases accordingly. The resultant ERI of the TE mode in the GERSW [Fig. 3(a)] and the TM mode in the RSW [not shown here], respectively, undergo a reduction and enhancement with the increased graphene number. For the GERSW, it leads to a larger variation in the modal characteristics for the TE mode if more graphene layers are used. However, the influence of the graphene number on the modal characteristics will be weakened if more graphene layers are involved so that the graphene layers on the top and bottom tend to have less interaction with the modal field. Therefore, for simplicity, we consider that the number of graphene layers is less than 7. For different graphene numbers, the width of the GERSW (*w*_g0_, *w*_g1_, *w*_g3_, *w*_g5_ and *w*_g7_) must be optimized to satisfy the phase-matching condition between the TE mode in the GERSW and the TM mode in the RSW [Fig. 3(a)]. In order to investigate the propagation property of the designed PSR, a three-dimensional eigenmode expansion (EME) method with the software Lumerical MODE Solutions has been employed to conduct the simulation. After that, the coupling length is optimized to obtain the maximum conversion efficiency for these five cases [see *L*_0_, *L*_1_, *L*_3_, *L*_5_ and *L*_7_ in Fig. 3(b)]. As shown in Fig. 3(c), with the optimized width of the GERSW (*w*_g7_) and the coupling length (*L*_7_), the TM input from the RSW is converted to the TE mode in the GERSW and efficiently outputs from the cross port, while the launched TE mode is barely influenced by the GERSW and directly outputs from the thru port. Because the modal characteristics of the TM mode in the GERSW would be negligibly influenced by using different numbers of graphene layers around *μ*_c_ = 0.5 eV [Fig. 3(d)], the wavelength dependence of the PSR for the TM input is almost unchanged [Fig. 3(e)]. It should be noted that the retrieved coupling length inevitably must deviate from the theoretical one, which induces that the maximum conversion efficiency does not occur at 1550 nm.

**Figure 4:**
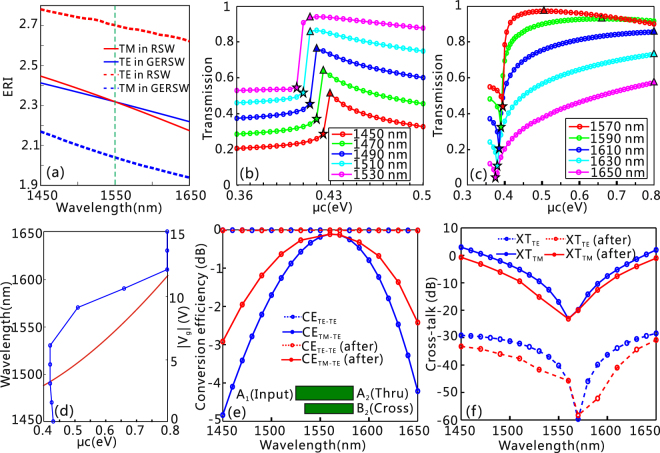
The influence of *μ*_c_ on the performance of the PSR. (**a**) The ERI for the TM mode in the RSW and TE mode in the GERSW as a function of wavelength. (**b,c**) When the TM mode is launched into the GERSW, the transmission of the converted TE mode at the cross port as a function of *μ*_c_ at (**b**) *λ* = 1450, 1470, 1490, 1510, and 1530 nm, and (**c**) *λ* = 1570, 1590, 1610, 1630, and 1650 nm. The star marks correspond to the peak positions for the Re(*n*) of the TE mode in the GERSW as shown in Fig. 1, and the triangle marks stand for the positions of the maximum transmission in (**b**) and (**c**). (**d**) The wavelength and applied voltage, ${V}_{g}$, versus *μ*_c_, at which the TM mode in the RSW can be efficiently converted to the TE mode in the GERSW. (**e**,**f**) Wavelength dependence of the PSR before and after optimization of *μ*_c_, (**e**) conversion efficiency *CE*_TE-TE_ and *CE*_TM-TE_, and (**f**) polarization cross-talk *XT*_TE_ and *XT*_TM_. The inset of (**e**) schematically shows the three ports.

Considering that the chemical potential of graphene could be tuned by bias voltage, we further investigate the influence of *μ*_c_ on the performance of the PSR. First, a PSR with seven layers of embedded graphene is studied as an example. All the structural parameters are the same as the above cases where the launched TM mode in the RSW could be efficiently converted to the TE mode in the GERSW at *λ* = 1550 nm. As for the TE mode input, an apparent phase-mismatching between the TE mode in the RSW and the TE and TM modes in the GERSW prevents light transferring from the RSW to the GERSW [Fig. 4(a)]. In other words, the TE mode launched from the RSW will directly output from the thru port with little interaction with the GERSW. We can see from Fig. 4(a) that decreasing (increasing) the operation wavelength will make the ERI of the TE mode in the GERSW smaller (larger) than that of the TM mode in the RSW. Therefore, it is not surprising that the change tendency of the conversion efficiency as a function of *μ*_c_ is different for the case of *λ* < 1550 nm [Fig. 4(b)] and λ > 1550 nm [Fig. 4(c)]. In the case of *λ* < 1550 nm (>1550 nm), the ERI of the TE mode in the GERSW can be increased (decreased) by tuning the chemical potential of graphene to make the phase-matching condition well satisfied; thus, the operation bandwidth of the PSR might be effectively extended. In the case of *λ* < 1550 nm, the ERI of the TE mode in the GERSW can be made closer to the ERI of the TM mode in the RSW by tuning *μ*_c_ from the triangle-mark positions to the star-mark positions. However, the significantly increased propagation loss [see Fig. 1(b)] would suppress the transmission of the TE mode. Therefore, the maximum transmission of the converted TE mode at the cross port is achieved at the triangle mark positions [Fig. 4(b)]. In the case of *λ* > 1550 nm [Fig. 4(c)], the maximum transmission could be achieved when *μ*_c_ < 0.8 eV for *λ* = 1570 and 1590 nm, and the maximum transmission for *λ* = 1610, 1630, and 1650 nm occurs at *μ*_c_ = 0.8 eV in our case. It can be highly expected that the transmission can be further enhanced if a much higher carrier density of graphene, associated with a larger chemical potential, is used. Consequently, to achieve the maximum transmission for the TE mode at the cross port, one must properly select the ERI and propagation loss for the TE mode in the GERSW.

The optimized value of *μ*_c_ as a function of wavelength, and *μ*_c_ versus ${V}_{g}$ are depicted in Fig. 4(d). We emphasize here the required ${V}_{g}$, associated with *μ*_*c*_ ranging from 0.4–0.8 eV, is below 11.7 V, which is comparable to that in graphene-based SOI modulators^13,14,22,23^. Figure 4(e) and (f) demonstrate that, after optimization of *μ*_c_ the device performance of the PSR has significantly improved as compared with that at *μ*_c_ = 0.5 eV. Here, for the TE mode input, the TE-TE conversion efficiency (*CE*_TE-TE_) at the through port A_2_ and the cross-talk (*XT*_TE_) at the cross port B_2_ are defined as $C{E}_{TE-TE}=10\,\mathrm{log}\,[P{(out)}_{TE}^{{A}_{2}}/(P{(in)}_{TE}^{{A}_{1}})]$ and $X{T}_{TE}=10\,\mathrm{log}\,[P{(out)}_{TE}^{{B}_{2}}/(P{(out)}_{TE}^{{A}_{2}})]$, respectively. For the TM mode input, the conversion efficiency (*CE*_TM-TE_) at the cross port B_2_ and the cross-talk at the thru port A_2_ are defined as $C{E}_{TM-TE}=10\,\mathrm{log}\,[P{(out)}_{TE}^{{B}_{2}}/(P{(in)}_{TM}^{{A}_{1}})]$ and $X{T}_{TM}=10\,\mathrm{log}\,[P{(out)}_{TM}^{{A}_{2}}/(P{(out)}_{TE}^{{B}_{2}})]$, respectively. As shown in Fig. 4(e) and (f), after optimizing the chemical potential of PSR, the bandwidth for *CE*_TM-TE_ (>−0.5 dB) has been extended from 58 nm (1533–1591 nm) to 86 nm (1516–1602 nm), while the cross-talks *XT*_TE_ and *XT*_TM_ have improved significantly in the wavelength range of interest. No matter what chemical potential of graphene is used, the *CE*_TE-TE_ is kept less than −0.02 dB over the whole studied spectral band, which can be attributed to the fact that the TE mode passes through the ADC with little influence due to the significant phase-mismatch.

**Figure 5:**
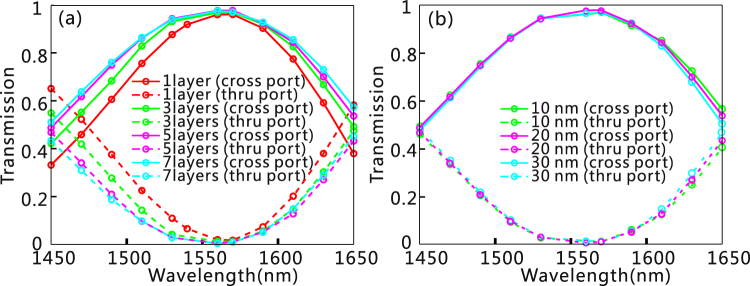
Wavelength dependence of the PSR with the TM mode input. (**a**) Wavelength dependence for different number of graphene layer (1, 3, 5 and 7). (**b**) Wavelength dependence for three different gap distances between every two adjacent graphene layers for the PSR with 7-layer graphene embedded. In each case, the chemical potential of graphene is optimized to make the phase-matching condition precisely satisfied for the ADC.

We further study the impact of the graphene number on the bandwidth broadening of the designed PSR. As shown in Fig. 5(a), more graphene layers leads to a broader bandwidth for the PSR. This is because the ERI has a larger tunability when more graphene layers are involved^18^. It should be noted that the electric field of the TE mode is strongest at the centre of the waveguide. As a result, the graphene layer in the middle has the largest contribution to the variation of the ERI, while the graphene layers on the upper and lower sides have the smaller influence on the ERI. This is why the growth rate of the operation bandwidth is decreased with an increase in graphene number [Table 1]. In addition, we have investigated the influence of the gap distance between every two adjacent graphene layers on the performance of the PSR [Fig. 5(b)]. For three different gap distances between every two adjacent graphene layers (10, 20, and 30 nm), the resultant device performance is almost kept unchanged, indicating that this design is very robust to the fabrication variation.

**Table 1:** Bandwidth of the PSR before and after the optimization of graphene’s chemical potential.

Number of graphene layer	Bandwidth of *CE*_TM-TE_ > −0.5 dB before optimizing *μ*_c_ (nm)	Bandwidth of *CE*_TM-TE_ > −0.5 dB after optimizing *μ*_c_ (nm)	*L*(μm)
0	54	—	10.3
1	54	61	10.4
3	57	77	10.5
5	59	84	10.8
7	58	86	11.1

Table 2 summarizes the bandwidths of several ADC-based PSRs from the latest literature and the present graphene-based PSR. Because the intrinsic phase-matching condition is required for a directional coupler, it is significantly limited to a narrow operating bandwidth for an ADC-based PSR^24,25^. The tapered ADC-based PSR has been recently proposed to overcome this limitation^7^, and the bandwidth is broadened as five times as large as the ADC-based PSRs^24,25^ but which is at the cost of a relatively large footprint. For the present graphene-based PSR, on one hand, the operation bandwidth can be four times larger than that in Refs^24,25^, while its length is several times smaller than that based on a tapered ADC-based PSR^7^. Our results here might offer a promising approach to construct a broadband and efficient PSR with an ultra-compact footprint. We have noted a recent study on a hybrid (de) multiplexer by combining mode, polarization, and wavelength together to increase the transmission capacity on a silicon platform^26^, where the polarization diversity circuit, comprised of a polarization beam splitter (PBS) and polarization rotation (PR), was used to address the issue of polarization dependence so that merely the TE mode was left for the arrayed-waveguide gratings (AWGs). Such a functionality with the combination of a PBS and PR, at least in principle, can be fulfilled with the proposed PSR, which has a larger working bandwidth that is required for the AWGs of 9 channels with the bandwidth of 9 × 3.2 nm (28.8 nm). In addition, more details regarding the performance of the PSR embedded with seven layers of graphene at 1550 nm have been listed in Table 2, including the insertion loss and extinction ratio. Compared with the simulation results of the PSR based on the taper-etched directional coupler, our proposal objects to comparable insertion loss with notably reduced device length.

**Table 2:** Comparison of ADC-based PSRs.

Reference	Gap (nm)	Criterion	Bandwidth (nm)	Length (μm)	Insertion loss	Extinction ratio	Result
Ref.^24^	150	CE_TM-TE_ > −1 dB	30	27	IL_TM-TE_ > −0.5 dB	>25 dB	Experiment
					IL_TE-TE_ > −0.3 dB		
Ref.^25^	100	CE_TM-TE_ > −1 dB	35	36.8	−0.6 dB (total)	12 dB	Experiment
Ref.^7^	200	CE_TM-TE_ > −0.5 dB	160	>80	IL_TM-TE_ > −0.09 dB	—	Simulation
					IL_TE-TE_ > −0.07 dB		
Our work (7-layer graphene)	150	CE_TM-TE_ > −0.5 dB	86	11.1	IL_TM-TE_ > −0.26 dB	>14 dB	Simulation
		CE_TM-TE_ > −1 dB	125		IL_TE-TE_ > −0.01 dB		

**Figure 6:**
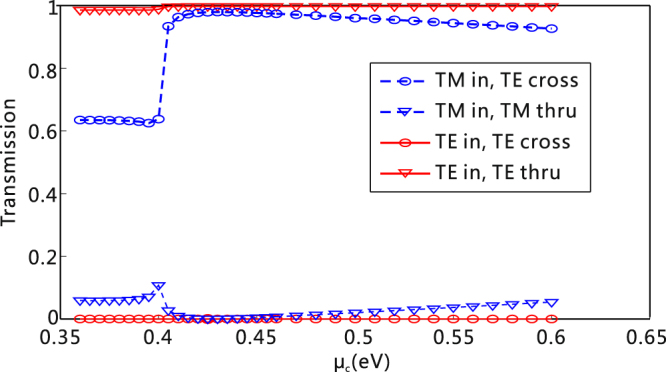
Influence of *μ*_c_ on the transmission of the PSR. Transmission at two output ports versus *μ*_*c*_ for the TE and TM input, respectively. The number of graphene layer is 5, and *λ* = 1550 nm.

Finally, it is worthy emphasizing, when the TM mode is launched, one can modulate the transmission of the TE mode at the cross port by tuning the chemical potential of graphene, while the transmission at the thru port almost stays unchanged [see the blue lines in Fig. 6]. Furthermore, when the TE mode is launched, the transmission of the TE mode both at the cross and thru ports is negligibly affected by the chemical potential of graphene [see the red lines in Fig. 6]. The variable splitting ratio power for the TM mode at the cross port may have various interesting applications in signal processing on the SOI platform. Finally, it is worth emphasizing that the graphene-based PSR might be very robust to the fabrication error because graphene affords the extra dimension of the effective refractive index tunability of waveguide mode, which offers the possibility of the construction of a feasible PSR without re-optimizing and refabricating the device structures.

## Conclusion

An ultra-compact PSR has been proposed and numerically demonstrated by utilizing an ADC consisting of a RSW and a GERSW. The ERI of the GERSW for the TE mode can be significantly changed by tuning the chemical potential of graphene. As a result, the phase-matching condition between the TM mode in the RSW and the TE mode in the GERSW can be well satisfied over a broad spectral band. The PSR embedded with seven layers of graphene and with a 11.1-μm coupling length and a 150-nm gap separation enables a high TM-to-TE conversion efficiency (>−0.5 dB) within a broad bandwidth (1516–1602 nm). Overall, this designed PSR presents a strong ability to address the issue of polarization dependence at 1550 nm with the TE-to-TE thru loss (>−0.01 dB), TM-to-TE polarization conversion loss (>−0.26 dB), and extinction ratio at the thru port (>14 dB).

## Methods

With the assumption of an e*xp*(−*iωt*) time dependence, the optical response of graphene can be characterized by surface conductivity (*σ*_g_), which is related to the chemical potential (*μ*_c_), using the Kubo formula^27^:

1 $$\begin{array}{ccc}{\sigma }_{g} & = & i\frac{{e}^{2}{k}_{B}T}{\pi {\hslash }^{2}(\omega +i{\tau }^{-1})}\,[\frac{{\mu }_{c}}{{k}_{B}T}+2\,\mathrm{ln}\,(\exp (-\frac{{\mu }_{c}}{{k}_{B}T})+1)]\\  &  & +i\frac{{e}^{2}}{4\pi \hslash }\,\mathrm{ln}\,[\frac{2|{\mu }_{c}|-\hslash (\omega +i{\tau }^{-1})}{2|{\mu }_{c}|+\hslash (\omega +i{\tau }^{-1})}]\end{array}$$

where *e* is the electron charge, *k*_B_ is the Boltzmann constant, *T* is temperature (=300 K), $\hslash$ is the reduced Planck’s constant, *ω* is the angular frequency, and *τ* is momentum relaxation (*τ* = 0.5 ps in this work). Because an electron mobility of 100000 cm^2^V^−1^s^−1^ has been experimentally verified in high-quality suspended graphene^28^, which leads to *τ* > 1.5 ps, the choice of *τ* = 0.5 ps here is rather conservative. Here, the graphene sheet is treated as an anisotropic material^29^. The out-of-plane permittivity of graphene, *ε*_g,⊥_, is equal to graphite (2.5). The in-plane permittivity of graphene, *ε*_g,∥_, can be retrieved as^30^

2 $${\varepsilon }_{g,//}=1+i\frac{{\sigma }_{g}{\eta }_{0}}{{k}_{0}{d}_{g}}$$

where *η*_0_ (≈377 Ω) and *k*_0_ are the impedance and wave vector in the air, respectively, and *d*_g_ (=0.34 nm) is the thickness of graphene. This equation has been extensively employed to characterize the optical property of graphene and estimate the electro-optical properties of graphene-based photoelectric devices both theoretically and experimentally^22,31^.

In the FDTD simulation, sufficiently small mesh girds for the silicon and graphene have been used to ensure the simulation accuracy. Four mesh grids are used to represent the thickness of the silicon layer between the adjacent graphene layers and each sheet of graphene. To precisely retrieve the device performance, different numbers of mesh grids have been tested in our modelling, and the present result indicates that the simulation date does not change if finer mesh grids are used. The perfectly matched layer-absorbing boundary condition is used at the boundaries of the computational window.
